# Impact of mitochondrial damage on tumor microenvironment and immune response: a comprehensive bibliometric analysis

**DOI:** 10.3389/fimmu.2024.1442027

**Published:** 2024-07-22

**Authors:** Yichun Xing, Yi Huang, Zhicheng Tang, Ying Lin, Yitong Zou, Yaqiang Huang, Zhaohui He, Qunxiong Huang, Jieying Wu

**Affiliations:** ^1^ Department of Obstetrics and Gynecology, Sun Yat-Sen Memorial Hospital, Sun Yat-Sen University, Guangzhou, Guangdong, China; ^2^ Department of Urology, Sun Yat-Sen Memorial Hospital, Sun Yat-Sen University, Guangzhou, Guangdong, China; ^3^ Department of Urology, The Third People’s Hospital of Chengdu/The Affiliated Hospital of Southwest Jiaotong University, Chengdu, Sichuan, China; ^4^ Department of Urology, the Eighth Affiliated Hospital of Sun Yat-Sen University, Guangzhou, Guangdong, China; ^5^ Department of Endocrinology, Sun Yat-Sen Memorial Hospital, Sun Yat-Sen University, Guangzhou, Guangdong, China; ^6^ Department of Urology, Zhongshan City People’s Hospital, Sunwen East Road, Zhongshan, Guangdong, China; ^7^ Department of Urology, the Third Affiliated Hospital of Sun Yat-Sen University, Guangzhou, Guangdong, China

**Keywords:** mitochondrial damage, tumor microenvironment, immune response, oxidative stress, bibliometric analysis

## Abstract

**Background:**

Mitochondrial damage contributes to apoptosis, oxidative stress, and inflammation, which collectively impact the immune system’s function and the tumor microenvironment (TME). These processes, in turn, influence tumor cell growth, migration, and response to treatment.

**Objective:**

We conducted a bibliometric analysis to elucidate the complex interactions between mitochondrial damage, the immune system, and the TME.

**Methods:**

Data were sourced from the Science Citation Index Core Collection (WoSCC) and analyzed using advanced tools like VOSviewer and Citespace. Our focus was on literature published between 1999 and 2023 concerning the interactions between mitochondrial damage and the TME, as well as immune responses to tumors. The analysis included regional contributions, journal influence, institutional collaborations, authorship, co-cited authors, and keyword citation bursts.

**Results:**

Our research encompassed 2,039 publications, revealing an increasing trend in annual output exploring the relationship between mitochondrial damage, TME dynamics, and immune responses. China, the United States, and South Korea emerged as the leading contributors. Prominent institutions included Institut National de la Santé et de la Recherche Médicale, University of Texas System, China Medical University, and Sun Yat-sen University. Key journals in this field are the International Journal of Molecular Sciences, Mitochondrion, and the European Journal of Pharmacology. Liang H and Wallace DC were identified as the most productive and co-cited authors, respectively. Keyword analysis highlighted the critical roles of inflammatory responses, oxidative stress, and the immune system in recent research.

**Conclusion:**

This bibliometric analysis provides a comprehensive overview of historical and current research trends, underscoring the pivotal role of mitochondrial damage in the TME and immune system.

## 1 Introduction

Cancer remains a leading cause of death and a significant health burden worldwide, drastically reducing life expectancy. Current projections indicate a potential 47% increase in the global cancer burden by 2040, positioning it as the foremost cause of premature death this century (1, 2). Tumor immunotherapy has shown significant promise and success across various cancer types in recent years. However, challenges related to treatment efficacy, organ-specific immune responses, and immune evasion highlight the necessity for deeper investigation in this field (3).

Mitochondria have attracted significant attention in tumor immunotherapy research, with alterations in mitochondrial dynamics being pivotal to tumor cell proliferation and migration (4). Beyond their traditional roles, mitochondria support tumors by enhancing energy production and biosynthesis, managing redox balance, and controlling cell death processes (5, 6). Moreover, mitochondria respond to cellular signals at the epigenetic level, influencing environmental stress and impacting tumor development, thereby emerging as promising new targets for cancer therapy (7). The immunoregulation and metabolic reprogramming of mitochondria remain crucial elements in cancer treatment. Interfering with these processes can suppress the growth and spread of tumor cells and improve the efficacy of immunotherapy (8).

The interplay between mitochondrial function and the immune system, particularly within the tumor microenvironment (TME), is critical. Mitochondrial metabolism influences various immunological processes, including tumor immune escape, which impacts tumor progression (9). Qiu et al. have suggested that mitochondrial-localized cyclic GMP-AMP synthase can inhibit peroxidation and ferroptosis by promoting the oligomerization of DRP1, thereby facilitating tumor immune escape (10). Additionally, Kuo et al. emphasize the crucial role of mitochondrial reactive oxygen species (mtROS) in the TME, regulating cancer cell proliferation and survival, modulating immune cell behavior to promote tumor immune evasion, and significantly impacting tumor development and resistance to treatment (11). Furthermore, genetic markers related to mitochondrial function can predict immunotherapy outcomes and immune cell infiltration in diseases like lung adenocarcinoma (12). While mitochondrial metabolism-related processes present promising new targets for enhancing tumor immunotherapy efficacy, and are closely linked with changes in the immune system and tumor microenvironment, the complex dual roles and influencing factors necessitate further elucidation of the underlying mechanisms.

Bibliometrics, a field based on the analysis of publication metadata, enables precise visualization and understanding of the current state of research in specific academic domains, helping stakeholders identify core research and future trends. This study employs bibliometric analysis to decipher the intricate web of literature and reveal how mitochondrial dynamics interact with the immune system and the TME. By building on historical literature developments and current academic discourse, this study aims to define and stimulate future research directions in line with the theme of the special issue.

## 2 Methods

### 2.1 Data sources

This study sourced its primary data from the Science Citation Index Expanded (SCI-Expanded) within the Web of Science Core Collection (WoSCC) by Clarivate Analytics, a highly regarded database in bibliometric research (13, 14). As the data used were publicly available, no ethical approval was necessary.

### 2.2 Search strategy

A comprehensive literature search was conducted on March 24, 2024, by two independent researchers to ensure thoroughness and accuracy. The search strategy involved the following terms: TS = ((mitochondrial OR chondriosome OR plastosome) NEAR/1 (damage OR dysfunction OR imbalance)) AND TS = (cancer* OR tumor* OR tumour* OR oncology OR neoplasm* OR carcinoma*) AND TS = (immune system OR immunological system OR T cell* OR B cell* OR natural killer cell* OR macrophage* OR dendritic cell*). From this search, 2039 publications were identified for further analysis. These publications, in English, spanned from 1999 to 2023 and included both articles and reviews.

### 2.3 Data processing and bibliometric analysis

The extracted data, including titles, keywords, abstracts, authors, institutions, and references, were meticulously downloaded and analyzed by two independent researchers. This dual analysis ensured the validity and reliability of the data. Microsoft Office Excel 2019 (Microsoft, Redmond, WA, USA) was employed for preliminary data examination.

For advanced bibliometric analysis, VOSviewer (version 1.6.16) and Citespace (version 6.3.1) were used. VOSviewer facilitated the visualization of the contributions by countries/regions, journals, authors, co-cited authors, and keywords. Citespace was instrumental in identifying key organizations and mapping the citation network to highlight 25 prominent keywords based on citation frequency (15, 16).

### 2.4 Analysis tools and techniques

#### 2.4.1 VOSviewer

Utilized to create visual representations of the bibliometric data, including network maps of authors, co-authors, and keywords. This tool helped in identifying major research trends and influential contributors within the field.

#### 2.4.2 Citespace

Used to perform co-citation analysis and detect emerging trends and pivotal research themes. It also helped in understanding the intellectual structure of the field by identifying key clusters and bursts in citation activity.

## 3 Results

### 3.1 Annual publication and citation trends

**Figure 1:**
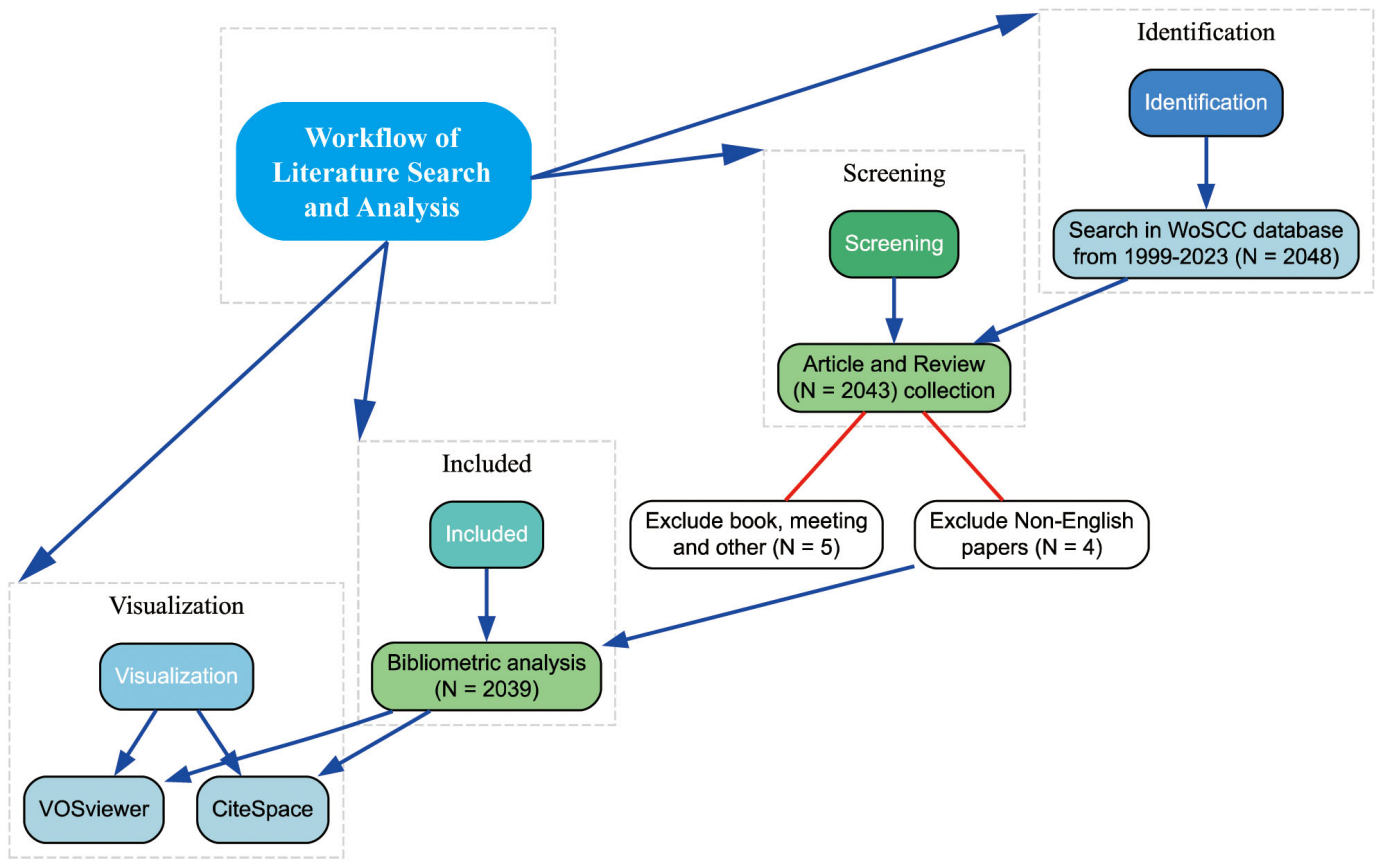
Flowchart detailing the inclusion process of studies for the bibliometric analysis.

**Figure 2:**
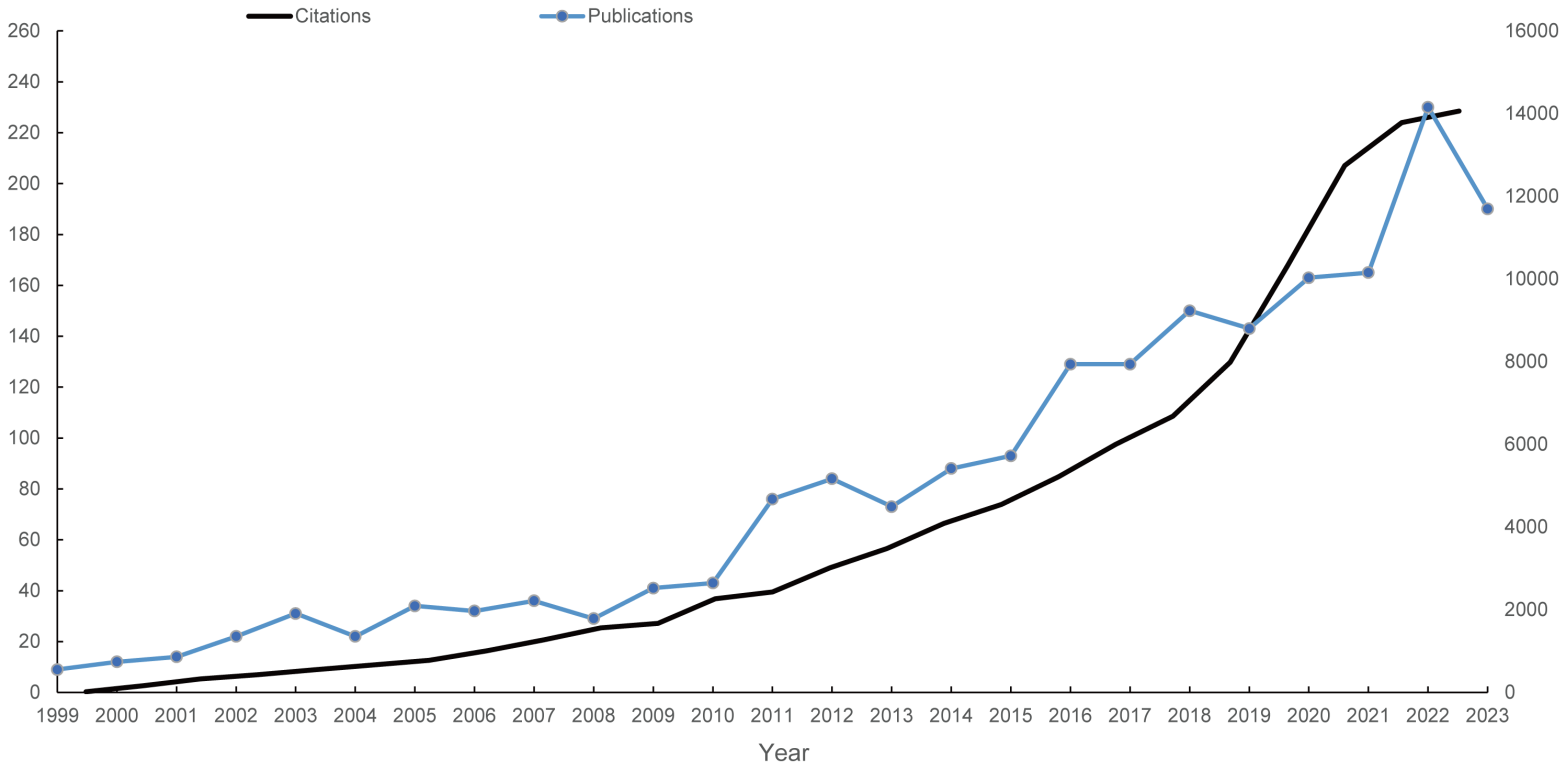
Annual publication and citation trends on mitochondrial dysfunction in the TME from 1999 to 2023. The left vertical axis indicates the annual publication count, while the right vertical axis represents the annual citation frequency. Blue dots represent the number of articles published each year, and the light black curve depicts the citation frequency over the same period.

Following the search strategy described earlier, 2039 papers spanning 1999 to 2023 were included in the analysis. **Figure 1** presents the flowchart detailing the inclusion process for these studies, highlighting the systematic approach taken to ensure comprehensive coverage of relevant literature. **Figure 2** illustrates the annual publication and citation trends, showing a consistent rise in both metrics over the years, particularly since 2018. On average, over 100 papers have been published annually, with citations peaking at over 14,000 in 2022. This increasing trend suggests a growing focus on the field, with more attention and resources being dedicated to research in this area.

### 3.2 Analysis of active countries and regions

**Figure 3:**
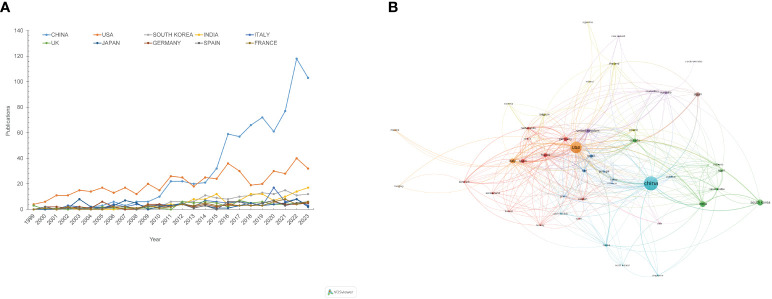
Visualization of annual publication trends and international collaborations from 1999 to 2023. **(A)** Annual publication trends for the top 10 contributing countries/regions. **(B)** Countries/regions collaboration network. Each node represents a country or region, with node size indicating their publication volume. Nodes of the same colour belong to the same category.

A total of 82 countries contributed to the literature, with the top 10 countries detailed in **Table 1**. China ranks first with 766 publications, followed by the United States (509), South Korea (138), India (126), Italy (98), and others. In terms of total citations and H-index, which indicate the average influence of articles, the United States ranks first with 38,653 citations and an H-index of 103, while China ranks last among the top 10 countries. The United Kingdom and France stand out with over 200 average citations per article, highlighting their significant research impact. **Figure 3A** shows the annual publication trends across the top 10 nations/regions, indicating a gradual increase, with a notable rise in contributions from Chinese scholars. Collaboration network analysis reveals strong connections between the US, Germany, France, China, and the UK (**Figure 3B**).

**Table 1:** Top 10 productive countries/regions concerning the research of mitochondrial dysfunction in tumor microenvironment.

Rank	Country/regions	Count	Total citations	H-index	Average citation per paper
1	China	766	20443	67	26.69
2	United States	509	38653	103	75.94
3	South Korea	138	4267	35	30.92
4	India	126	5063	36	40.18
5	Italy	98	7116	45	72.61
6	United Kingdom	78	19324	39	247.74
7	Japen	72	2736	29	38
8	Germany	70	13614	32	194.49
9	Spain	66	13131	34	48.59
10	France	65	14079	32	216.6

### 3.3 Active institutions and journals analysis

**Figure 4:**
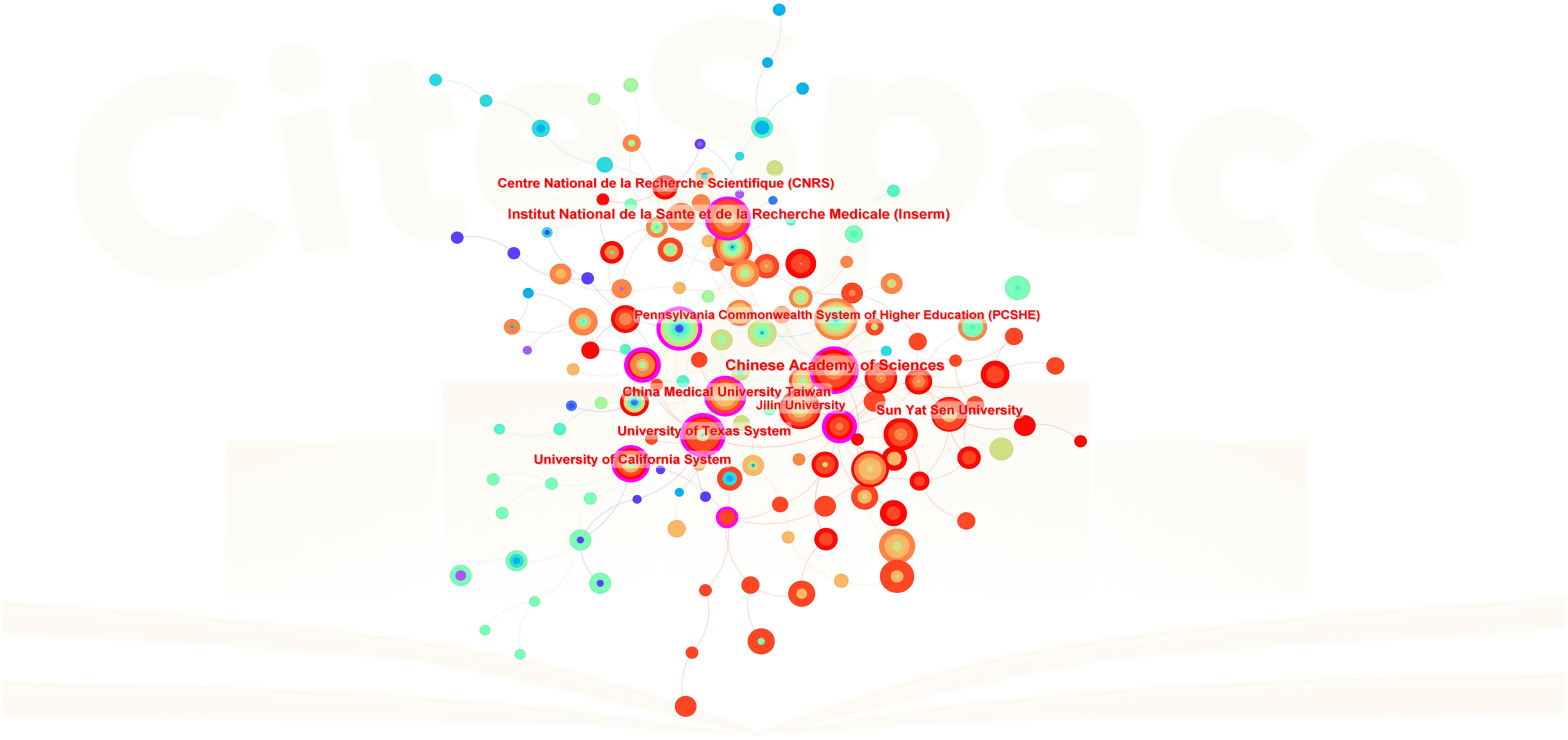
Inter-institutional collaboration network. Each node represents an institution, with node size proportional to the volume of publications contributed to the field of mitochondrial dysfunction in the TME. Lines between institutions represent collaborative relationships.

**Figure 5:**
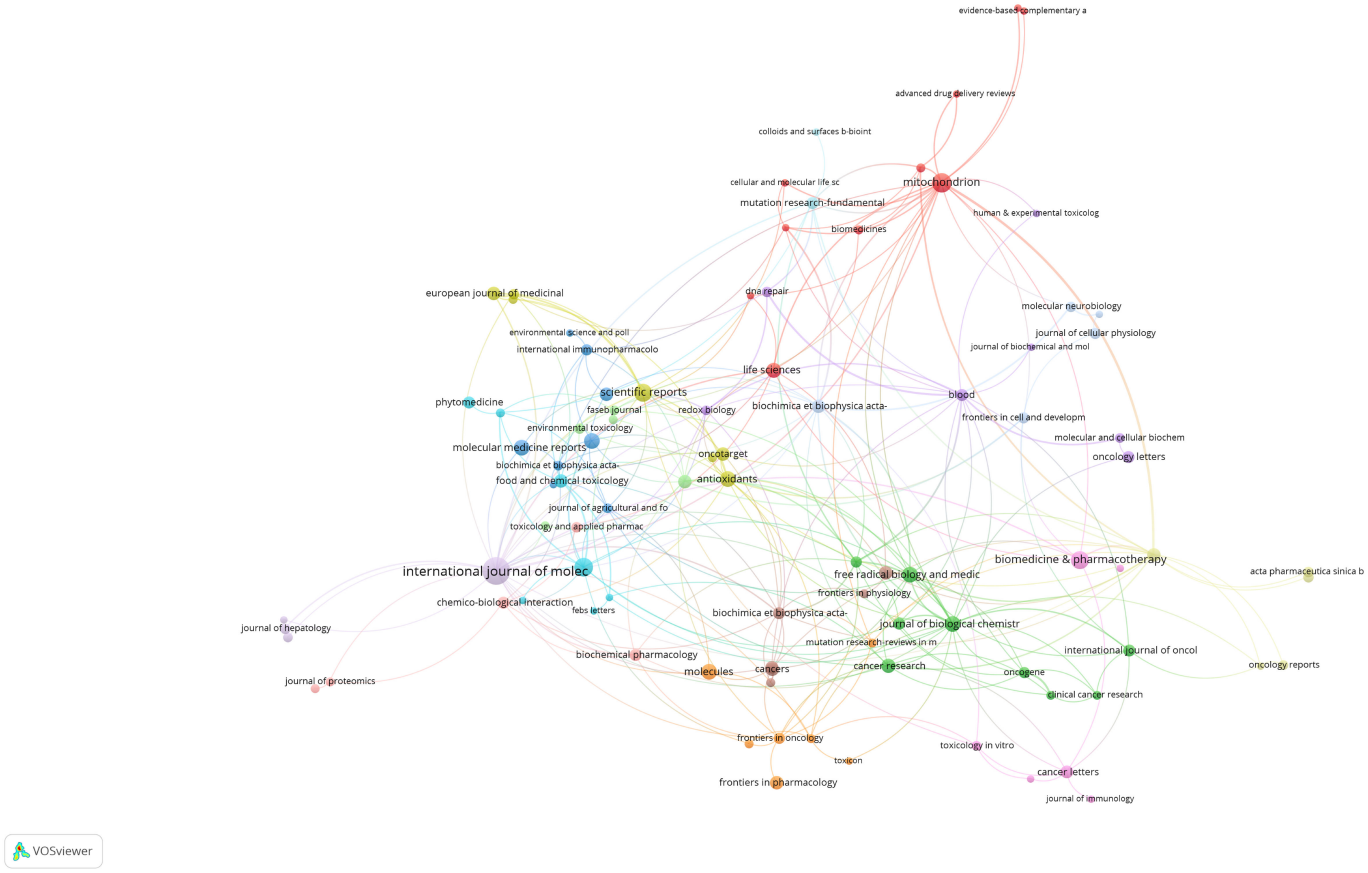
Journal collaboration network. Each node in this network diagram represents a scientific journal. Node size is proportional to the volume of publications contributed by that journal to the field of mitochondrial dysfunction in the TME. Lines connecting the nodes illustrate collaborations between journals, with thicker lines indicating more frequent cross-citations or shared authorship.

**Table 2** lists the top 10 contributing institutions, with the Chinese Academy of Sciences leading with 44 papers, followed by the Institut National de la Santé et de la Recherche Médicale (37), University of Texas System (31), China Medical University (31), and Sun Yat-sen University (31). The collaboration network map shows robust cooperation within Chinese and French institutions but weaker international collaboration. The Pennsylvania Commonwealth System acts as a bridge in international collaborations (**Figure 4**). The top 10 contributing journals are listed in **Table 3**, with the International Journal of Molecular Sciences leading with 59 papers, accounting for 22.7% of the top 10 journals’ combined total, followed by Mitochondrion (11.5%) and others. According to the 2023 Journal Citation Reports, four of the top 10 journals have impact factors exceeding 5. The collaboration analysis also indicates close connections between journals (**Figure 5**).

**Table 2:** Top 10 institutes in the publications concerning the research of mitochondrial dysfunction in tumor microenvironment.

Rank	Institutions	Countries/regions	Count	Centrality
1	Chinese Academy of Sciences	China	44	0.14
2	Institut National de la Sante et de la Recherche Medicale	France	37	0.16
3	University of Texas System	United States	31	0.31
4	China Medical University	China	31	0.12
5	Sun Yat Sen University	China	31	0.06
6	University of California System	USA	30	0.12
7	Centre National de la Recherche Scientifique	France	26	0.02
8	Pennsylvania Commonwealth System	USA	24	0.03
9	Jilin University	China	23	0.04
10	Harvard University	USA	22	0.08

**Table 3:** Top 10 Journals in the publications concerning the research of mitochondrial dysfunction in tumor microenvironment.

Rank	Journal title	Countries	Count	IF (2023)	JCR	Total citations
1	International journal of molecular sciences	United States	59	5.6	Q1	2012
2	Mitochondrion	England	30	4.4	Q1	3082
3	European journal of pharmacology	Netherlands	27	5.0	Q1	1002
4	Biomedicine & pharmacotherapy	France	25	7.5	Q1	801
5	Scientific reports	England	24	4.6	Q2	544
6	Plos one	United States	19	3.7	Q2	723
7	Molecules	Switzerland	19	4.6	Q2	475
8	Molecular medicine reports	Greece	19	3.4	Q3	417
9	Journal of biological chemistry	United States	19	4.8	Q2	1926
10	Free radical biology and medicine	United States	19	7.4	Q1	1905

### 3.4 Analysis of authors and co-cited authors

**Figure 6:**
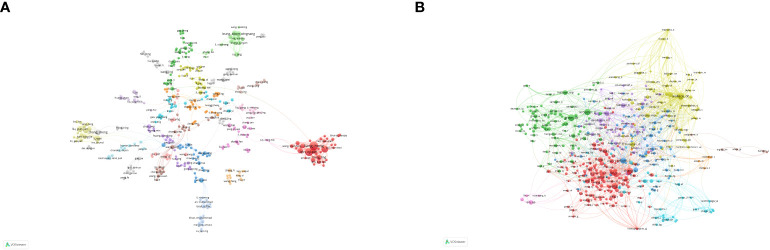
Analysis of authors and co-cited authors. **(A)** Visualization map of author analysis. **(B)** Visualization map of co-cited author analysis. Each node represents an author or co-cited author, with node size indicating their citation count or document volume. Lines between authors represent collaborative relationships.

Co-citation analysis, which shows a relationship between authors or papers cited together, is widely used to dissect a discipline’s historical background, current status, and future prospects. The top three authors by paper output are Liang H, Qin Q, and Liu Y, with 9 of the top 10 being Chinese scholars. **Figure 6A** illustrates author collaboration, showing strong cooperation within nations but weaker international ties. Visualizing co-cited authors reveals that Wallace D, Wang Y, and Green D are prominent in the field, while Galluzzi L and others play a notable role in fostering international collaboration (**Figure 6B**).

### 3.5 Keyword analysis

**Figure 7:**
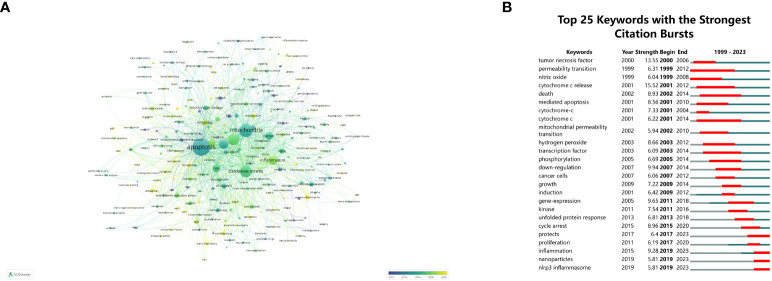
Keyword visualization and analysis. **(A)** Network visualization of keywords using VOSviewer, where node sizes increase with keyword frequency. **(B)** The 25 most frequently cited keywords relevant to mitochondrial dysfunction in the TME. The term “strength” refers to the connection intensity between two nodes, as determined by the software.

Keyword analysis statistically summarizes literature keywords, directly showing the core themes of articles in a specific field and reflecting research hotspots. Early research focused on mitochondria, apoptosis, cancer, oxidative stress, and inflammation, while recent studies have mentioned ferroptosis, necroptosis, and neuroinflammation, indicating potential research value (**Figure 7A**). The 25 most-cited keywords are listed in **Figure 7B**. Early keywords include tumor necrosis factor (13.55), permeability transition (6.31), nitric oxide (6.04), cytochrome c release (15.52), death (8.93), and mediated apoptosis (8.56), with permeability transition having the longest duration. Recent hot keywords include inflammation (9.28), cycle arrest (8.96), unfolded protein response (6.81), and protection (6.40).

### 3.6 Mechanistic insights

**Figure 8:**
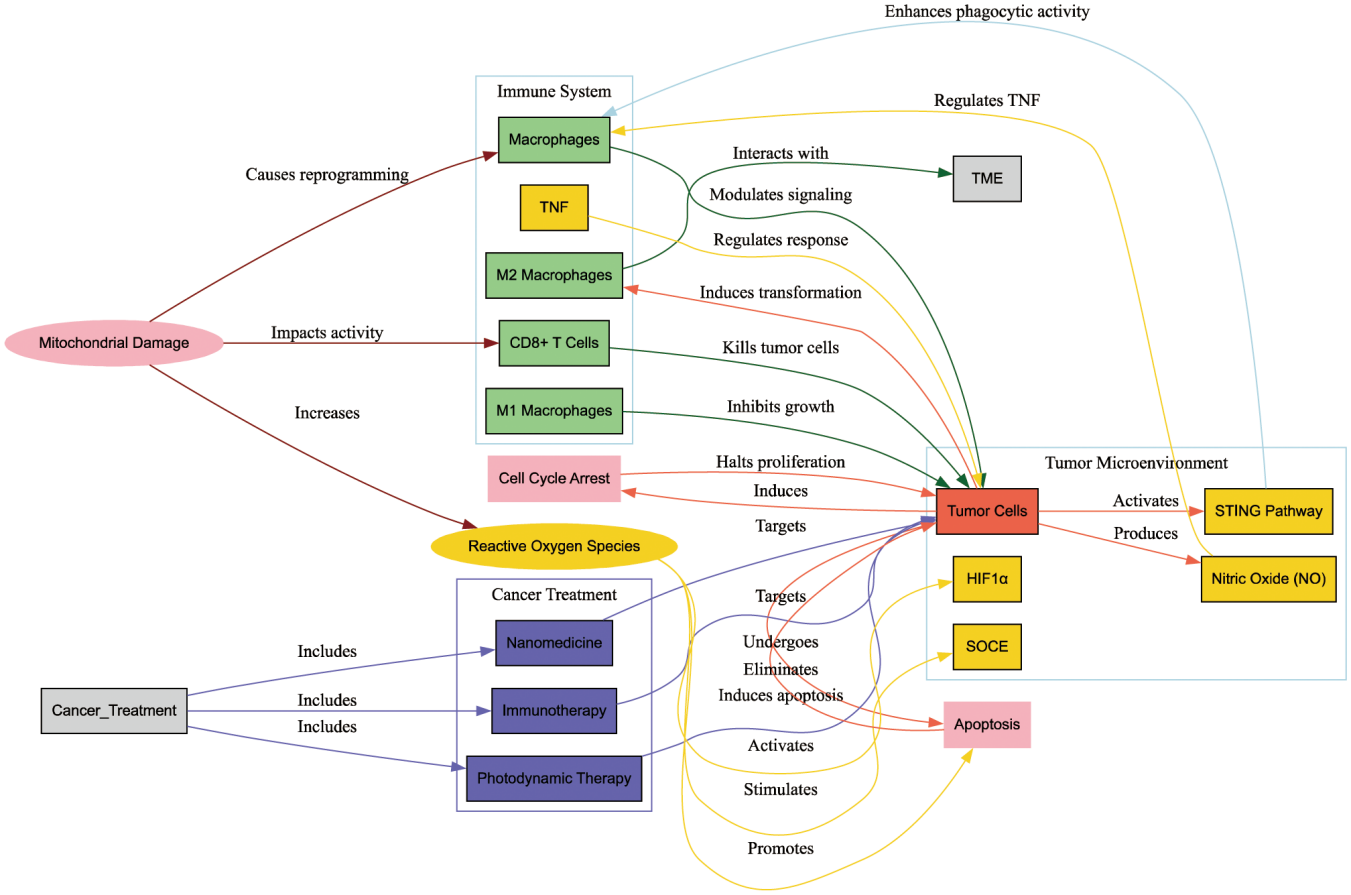
Interactions between mitochondrial damage, the immune system, the tumor microenvironment (TME), and cancer treatments. This diagram illustrates how mitochondrial damage influences CD8+ T cells and macrophages, leading to increased reactive oxygen species (ROS) production, cell cycle arrest, and apoptosis in tumor cells. Cancer treatments such as photodynamic therapy, nanomedicine, and immunotherapy target these interactions to enhance antitumor responses and inhibit tumor growth. The diagram underscores the critical role of mitochondrial damage in modulating immune responses and tumor progression, offering insights for improved cancer therapies.

**Figure 8** illustrates the interactions between mitochondrial damage, the immune system, the tumor microenvironment (TME), and cancer treatments. Mitochondrial damage impacts CD8+ T cells and macrophages, leading to increased ROS production, cell cycle arrest, and apoptosis in tumor cells. Cancer treatments such as photodynamic therapy, nanomedicine, and immunotherapy target these interactions to enhance antitumor responses and inhibit tumor growth. The diagram highlights the critical role of mitochondrial damage in modulating immune responses and tumor progression, providing insights for improved cancer therapies.

## 4 Discussion

### 4.1 General information

Cancer remains a leading cause of death globally. Despite advances in therapies, the complex role of mitochondria in tumor progression challenges treatment effectiveness and patient outcomes. This article provides an overview of key issues in this area, aiming to enhance researchers’ understanding of mitochondrial functions in the immune system and TME, and guide them through the extensive literature to identify promising research directions. Our bibliometric analysis highlights the surge in interest and insights into mitochondrial mechanisms, emphasizing their crucial role in cancer research.

The results of this research emphasize China’s prolific research output in this field, demonstrating rapid growth, while the United States excels in total citations and the H-index, indicating influential research contributions. Among the top ten most productive universities, four are Chinese, four are American, and two are French. Analysis of cooperation between countries/regions suggests close domestic connections within the United States, France, China, and the United Kingdom, and highlights global cooperation efforts on this topic.

Data visualization of author contributions highlights significant advancements in this field, particularly from authors like Liang H, Qin Q, and Liu L. Liang H and Qin Q collaborated on developing new anticancer Pt (II) complexes targeting telomerases, which induce mitochondrial dysfunction and apoptosis in bladder cancer cells, effectively suppressing tumors (17). Liu L and his team are at the forefront of cancer immunotherapy, exploring novel nanomaterials and photodynamic therapies. They discovered that nitric oxide can inhibit oxidative phosphorylation, mitigating the adverse effects of mitochondrial damage on macrophages and restoring their anticancer immune function (18). Our research identifies emerging hotspots in mitochondrial-related tumor immunology, focusing on apoptosis activation, cell cycle regulation, and cutting-edge nanotechnologies such as “tumor necrosis factor”. “permeability transition”, “nitric oxide”, “nanoparticles” and “nlrp3 inflammasome”. These findings underline the critical role of mitochondrial damage in cancer progression and its potential as a therapeutic target.

Drawing from the bibliometric analysis provided, this research is grounded in previous studies, delineates the current research landscape, and offers future direction and perspectives. This approach significantly aids in enhancing our comprehension of the interactions between mitochondrial damage, the immune system, and the tumor microenvironment, thereby fostering improvements in cancer treatment strategies.

### 4.2 The interaction mechanism of mitochondrial damage with the immune system and tumor microenvironment

The immune system is crucial for preventing cancer progression by recognizing and eliminating malignant cells. Dysregulation within this system often stems from immunosuppressive cells in the TME (19). Immunotherapies targeting the immune system and the TME have become a cornerstone of cancer treatment. These include immune checkpoint inhibitors targeting CTLA-4, PD-1, and PD-L1, therapeutic cancer vaccines like Sipuleucel-T, bi-specific T cell engagers (BiTEs), and chimeric antigen receptor T-cells (CAR-T) (19, 20). Despite progress, several challenges impede the broader efficacy of immunotherapy, including patient response heterogeneity, novel antigen development, suitable delivery systems for cancer vaccines, and limited CAR-T cell therapy effectiveness in solid tumors. Thus, deeper investigation into the fundamental mechanisms governing these phenomena is urgently needed to enhance the therapeutic potential of immunotherapy (21).

### 4.3 Mitochondrial damage and immune cells

Mitochondrial damage and its interaction with the immune system remain a hot topic, particularly in studies involving T cells and macrophages. CD8+ T cells play a pivotal role in the body’s antitumor processes, releasing perforin to kill tumor cells and secreting cytokines like TNF to regulate the antitumor response, with mitochondria playing a multifunctional role throughout (22). Mitochondria aid in chemotaxis, gather calcium ion waves to boost activation, and release mitochondrial reactive oxygen species (mROS), vital for maintaining cell survival and functionality, thus significantly impacting T cell antitumor activity (23). Advances in omics technologies have revealed that the transfer of mitochondria through tunneling nanotubes from T cells to cancer cells is linked to tumor proliferation and patient prognosis (24).

### 4.4 Macrophage polarization and mitochondrial function

Macrophages, as phagocytic immune cells, exhibit significant heterogeneity and are critical in maintaining physiological stability and contributing to cancer progression (25). M2 polarized macrophages aid in tissue repair and immune tolerance, ultimately leading to tumor suppression. Under mitochondrial guidance, macrophages undergo immune metabolic reprogramming and fatty acid oxidation, becoming tumor-associated macrophages (TAMs) that interact with the TME, significantly impacting cancer cell signaling and proliferation (25, 26). High levels of nitric oxide (NO) produced by cancer cells may regulate the expression of tumor necrosis factors by changing the mitochondrial outer membrane permeability, leading to mitochondrial damage and affecting tumor cell apoptosis. Mitochondrial dysfunction in M2 macrophages can cause metabolic reprogramming and suppress antitumor activities like phagocytosis. *In vivo* models have shown that malignant plasma cells can release mtDNA into the myeloma TME, activating marrow macrophages via the STING signaling pathway, enhancing phagocytic activity, and impacting tumor burden (27). Yao Lu and colleagues have developed novel copper complexes targeting mitochondrial metabolism, such as copper (II) bis(diethyldithiocarbamate) (CuET), which amplify immunogenic death in breast cancer cells, release damage-associated molecular patterns, and effectively induce M1 polarization and macrophage migration, contributing to their anticancer effects (28).

### 4.5 Tumor microenvironment and mitochondrial dynamics

As the view of tumor progression as a dynamic ecosystem gains acceptance, the TME, comprising fibroblasts, immune cells, vascular systems, and other components, is receiving increasing attention. It is confirmed to be crucial for tumor progression and treatment, with potential for further exploration through spatial transcriptomics to improve cancer diagnostics and therapy (29). Recent studies highlight mitochondria as pivotal targets within the TME, influencing the behavior of cancer and stromal cells under conditions such as tumor hypoxia, mitochondrial oxidative phosphorylation (OXPHOS), and tricarboxylic acid cycle processes. These processes contribute to metabolic reprogramming and extracellular acidification in cancer cells, with small molecule inhibitors of mitochondrial OXPHOS offering a method to disrupt these adaptations (30). Oxidative stress, characterized by excessive reactive oxygen species (ROS) production, plays a crucial role in modulating cancer signaling and the TME. Elevated ROS from mitochondrial dysfunction can promote cancer cell proliferation by activating HIF1α and stimulating calcium signaling through STIM1 and SOCE. It can also drive mitochondrial autophagy, influence antigen-antibody complex formation, suppress T-cell responses, and affect macrophage polarization (31). As a major endogenous source, the high levels of ROS produced by mitochondrial dysfunction may contribute to an immunosuppressive tumor microenvironment. Firstly, it may promote cancer cell proliferation through the activation of HIF1α, which leads to STIM1 puncta formation and SOCE activation (32), Secondly, it could activate mitochondrial autophagy, depending on the heterogeneity of the tumor. Then, by affecting the formation of antigen-antibody complexes, it may suppress T-cell responses and regulate macrophage polarization (11).

### 4.6 Cell cycle arrest and mitochondrial dysfunction

Cell cycle arrest is another critical avenue through which mitochondria can impact tumor progression. Research has demonstrated that 3-bromopyruvate (3-BP) disrupts mitochondrial membrane potential, boosts immune infiltration, and induces tumor cell cycle arrest in the TME (33). Similarly, novel dyes targeting mitochondria in melanoma cells have been shown to induce G0/G1 cell cycle arrest by targeting the E2F/Cyclin/CDK pathway, initiating tumor cell apoptosis, and inhibiting their growth (34). Additionally, chlorpromazine induces G2/M cell cycle arrest and mitochondrial-dependent apoptosis, effectively suppressing the growth and metastasis of colorectal cancer in animal models (35).

### 4.7 Emerging therapies and mitochondrial targets

Despite recent successes in cancer immunotherapy, particularly with checkpoint inhibitors, challenges such as patient variability and tumor immune evasion persist. The interplay between mitochondrial damage, the immune system, and the TME is crucial for tumor immune surveillance. Targeting these interactions offers promising avenues for enhancing cancer treatment. Photodynamic therapy (PDT) is gaining traction as a novel treatment modality. Rhenium-guanidine complexes have been shown to act as effective photosensitizers, triggering apoptosis, mitochondrial dysfunction, and cell cycle arrest (36). Advancements in nanotechnology further complement these therapies. Liposomal nanomedicines encapsulating doxorubicin and chlorin e6 have enhanced PDT efficacy by reshaping the heterogeneous TME and boosting tumor immunogenicity (37). Additionally, CaO2@CuSMnO2@HA (CCMH) nanocomposites induce the release of damaged mitochondrial DNA from tumor cells, prompting macrophage transformation to the M1 phenotype, reshaping the TME, and augmenting the effectiveness of immunogenic cell death inducers in breast and colorectal cancer models (38). Similarly, zero-valent iron nanoparticles (ZVI-NP) induce mitochondrial damage and ferroptosis in lung cancer cells while modulating macrophage and CD8+ cell transformation, improving anticancer efficacy by altering the TME (39).

## 5 Limitation

Primarily, this literature review was restricted to English-language publications, potentially omitting significant contributions in other languages. Furthermore, the delayed updates of Web of Science keywords and the complexities inherent in collecting citation data mean that recent developments, especially novel treatments targeting mitochondrial sites, might not be fully captured in our bibliometric analysis.

## 6 Conclusion

This bibliometric review highlights the dynamic and evolving research on mitochondrial damage in cancer, particularly its interactions with the immune system and the tumor microenvironment. Our study reveals a shift from traditional mitochondrial apoptosis signaling to oxidative stress and immune modulation, emphasizing the roles of CD8+ cells and macrophages. These insights are crucial for developing new therapeutic targets and improving existing treatments, ultimately aiming to enhance cancer prognosis. By understanding and targeting mitochondrial functions and their impact on the immune system, we can pave the way for more effective cancer therapies. Continuous research in this area holds promise for significant advancements in cancer treatment strategies, contributing to better patient outcomes and a deeper understanding of cancer biology.

## Data availability statement

The raw data supporting the conclusions of this article will be made available by the authors, without undue reservation.

## Author contributions

YX: Writing – review & editing, Formal analysis, Conceptualization. YiH: Writing – review & editing, Formal analysis, Conceptualization. ZT: Writing – review & editing, Formal analysis, Conceptualization. YL: Writing – original draft, Visualization, Methodology, Data curation. YZ: Writing – original draft, Visualization, Methodology, Data curation. YaH: Writing – original draft, Visualization, Methodology, Data curation. ZH: Writing – review & editing, Supervision. QH: Writing – review & editing, Supervision. JW: Writing – review & editing, Supervision.
